# Validity and reliability of the Turkish version of the transition of primiparas becoming mothers scale

**DOI:** 10.1186/s12884-024-06438-7

**Published:** 2024-04-11

**Authors:** Zila Özlem Kirbaş, Elif Odabaşi Aktaş, Hava Özkan

**Affiliations:** 1 Department of Nursing, Faculty of Health Sciences, Bayburt University, Bayburt, Türkiye; 2 Department of Midwifery, Faculty of Health Sciences, Bayburt University, Bayburt, Türkiye; 3 Department of Midwifery, Faculty of Health Sciences, Atatürk University, Erzurum, Türkiye

**Keywords:** Transition, Primiparas, Turkish, Translation, Psychometrics, Validation

## Abstract

**Background:**

The transition to motherhood is an important life event in a woman’s life and represents an important developmental process that brings physical, psychological and social changes to gain a new role. However, research on the transition to motherhood in Turkish society is scarce. There is a need for a comprehensive, practical and reliable tool to evaluate the transition to motherhood in primiparous mothers. This study evaluated the reliability and validity of the Turkish version of the Transition of Primiparous Becoming Mothers Scale (TMP-S) to evaluate the transition process of primiparous mothers to motherhood.

**Methods:**

This methodological research was carried out in obstetrics and gynecology outpatient clinics, pediatric outpatient clinics, and family health centers of a hospital in Türkiye. The sample consisted of primiparous mothers of 0 to 6- month-old babies who visited clinics and family health centers for routine postnatal examinations (n ​​= 305). After evaluating the language equivalence and content validity of the scale, test-retest reliability, internal consistency and construct validity were examined. Factor analysis, Pearson’s correlation, retest reliability, and Cronbach’s alpha were employed to evaluate structural validity and reliability.

**Results:**

The final TPM-S had two dimensions with 25 items. Exploratory factor analysis revealed a two-factor solution, which accounted for 59.276% of the variance. Confirmatory factor analysis showed that the model fit of the two-factor model also reached a satisfactory model ft after modification. The comparative fit index was 0.894, the Tucker‒Lewis index was 0.882, and the root mean square error of approximation was 0.079. The content validity index of the scale ranged from 0.56 ~ 0.77. The Cronbach’s alpha coefficient was 0.93 for the total scale, and the test–retest reliability was 0.96.

**Conclusions:**

It is a valid and reliable tool for evaluating the transition to motherhood among primiparous mothers of 0 to 6 month-old babies in Türkiye. Turkish researchers and healthcare professionals can routinely apply this measurement tool to primiparous mothers in the first six months after birth to evaluate their transition to motherhood.

## Introduction

The transition to motherhood is one of women’s most significant developmental life events. Becoming a mother refers to transitioning from a known situation to an unknown status and a new role. Transitioning to motherhood requires reinterpreting goals, behaviors, and responsibilities to obtain new meanings [1–3]. The transition to motherhood, which begins during pregnancy, continues in the postnatal period [4]. The postpartum period is when adaptation to parenthood and secure attachment with the newborn baby can develop [5]. Moving toward a new normal phase, the woman begins to structure her motherhood to suit herself and her family according to her future goals. Adapted to changing relationships with spouses, family, and friends. Many cognitive restructurings occur as she learns her baby’s cues and what is best for her baby and adapts to her new reality [6].

Gradually, women in the process of accessing the maternal role learn the behaviors expected from the maternal role. She imitates the maternal performances she observes by following the rules of motherhood and the guidance of other mothers. Thus, she develops a unique behavioral pattern and gains self-confidence and competence in her maternal role [7]. Both the mother’s health and well-being are at risk during this period, as are her baby’s well-being and the stability of her family. Some special conditions, such as complex and lengthy postpartum recovery and newborn admission to the intensive care unit, may cause disruptions in the transition to motherhood. Nurses and midwives need to have a basic understanding of the motherhood transition to facilitate the process for mothers and babies at risk [4].

The stage after birth involves becoming familiar with the maternal role and learning to care for a child, where childcare becomes part of daily life [4]. Slade et al. [8] stated that the process of becoming a mother causes many essential and permanent changes in pregnant women’s lives; therefore, pregnant women may have difficulty accepting the role of motherhood. Study results have shown that primiparous women and those who are not ready for motherhood experience intense stress during this period [9, 10]. Women with multiple pregnancies may also experience problems adapting to motherhood because they experience more anxiety [9]. The mothers’ perceptions of pregnant women who are hospitalized due to any risk are also negatively affected [11]. Shorey et al. [12] stated that primiparous women’s perception of parental competence is lower than that of multiparous women.

The transition to motherhood and the maternal role is affected by the social and cultural values ​​of the individual and the society in which he or she lives. In particular, the value society places on the status of motherhood significantly affects the transition to motherhood [13]. In Turkish society, women perceive motherhood as the most essential duty. This task constitutes a large part of women’s daily lives. However, women may sometimes experience inadequacies in their motherhood roles due to their individual, physical, and psychological characteristics and social, cultural, and economic situations [14]. This situation can affect a child’s health, growth, and development. According to motherhood theory, nurses and midwives are health professionals with essential roles in women’s transition to motherhood and their adaptation to maternal roles [15]. Therefore, nurses and midwives must be able to identify and measure progress in the transition to motherhood to provide adequate physical, psychosocial, and self-care support to mothers. For this purpose, Katou et al. [4] developed a measurement tool to determine the progress of Japanese primiparas in the transition to motherhood. However, in Türkiye, there is currently no measurement tool that can be used to determine the actual status of adjusting to life in the role of mother.

Today, measurement tools are being developed regarding the acquisition of the postpartum maternal role in Türkiye and various countries. However, since the conditions related to motherhood differ in each country, applying a measurement tool initially developed in other countries to the Turkish population takes time and effort. In addition, existing tools measure confidence, capacity, compassion, level of satisfaction and sense of self-efficacy as the state of acquisition of the maternal role [16–20], and instruments have been developed as scales to determine whether the maternal role has been achieved or to assess poor postpartum physical and mental states [20–24]. However, there is currently no tool for determining the actual situation of adjusting to life through the role of motherhood in Turkey. To provide support to the Turkish primipara, we believe that it is imperative to understand the actual process of adjusting to life in the motherhood role during the transition to motherhood. No study of the adaptation, validity, or reliability of this scale, which was developed in Japan, has been conducted in another culture. This study is the first cross-cultural adaptation, validity, and reliability study of the scale. Therefore, our study was carried out via face-to-face interviews in gynecology and obstetrics outpatient clinics, pediatric outpatient clinics and family health centers of a hospital in Türkiye between November 2022 and May 2023.

To support mothers, it is imperative to understand the process of adjusting to life in the mother’s role as a transition to becoming a mother. Therefore, in this study, we aimed to develop a Turkish version of the measurement tool developed to determine the progress of Japanese primiparas in the transition to motherhood and to examine its psychometric properties and factor structure to shed light on the transition of Turkish primiparas in the process of becoming mothers.

## Methods

### Aim and design

This study is a methodological study to test the validity and reliability of the TPM-S in the Turkish context. The scale aims to determine the care self-efficacy levels of pediatric nurses. The study findings were presented following the guidelines outlined in the STROBE.

### Participants and procedures

This methodological research was carried out in obstetrics and gynecology outpatient clinics, pediatric outpatient clinics, and family health centers of a hospital in Türkiye between November 2022 and May 2023. The sample consisted of primiparous mothers of 0 to 6-month-old babies who visited clinics and family health centers for routine postnatal examinations. Since regular checks in Türkiye are performed by hospitals and family health centers (FHCs), data were collected in these centers for ease of access to mothers. When adapting a measurement tool to another culture, it is recommended to include at least 5–10 times as many participants as the number of items in the measurement tool [25]. However, Hogarty et al. [26] suggested that this ratio should be 20:1. In scale psychometric studies, it is recommended that the number of items forming the scale be 5–20 times greater, the factor structure be stable, and the sample size be at least 300 participants so that the results can be generalized [27]. Accordingly, this study’s sample size was 300 (30 × 10 = 300), with at least ten primiparous mothers per item. Primiparous mothers of 0 to 6-month-old babies who came to the clinic and FHCs between the data collection dates were included in the study by a random sampling method. The study included 305 primiparous mothers. Power analysis was performed to determine whether the number of samples was sufficient to detect significant differences. Power analysis revealed a power of 100% with an effect size of 0.7 (α = 0.05). The results showed that the sample size was sufficient.

The inclusion criteria for primiparous mothers were as follows: having a live birth at the age of 18 or over (without a baby with a congenital anomaly, a premature baby, a low birth weight baby, or a multiple birth), the mother’s verbal declaration that she was not diagnosed with any mental disorder, and being open to communication to be a primiparous mother between 1 and 6 months postnatally. Mothers who did not want to participate in the study were excluded from the study. When mothers underwent routine examination or vaccination of their babies in the hospitals and centers where the research was conducted, they were informed about the purpose and procedure of the research before participating in the study, and informed consent was obtained from those who agreed to participate. After approval, the mothers completed the data collection forms individually to reduce the impact of the research on the mothers. Explanations were given when participants had questions about the study.

### Measures

Data were collected with a sociodemographic survey and the Transition of Primiparas Becoming Mothers scale.

### Sociodemographic questionnaire

The researchers created the survey by reviewing the relevant literature. It consists of sociodemographic information such as maternal age, educational background, working status, household income, place of residence, family type, and postnatal month and type of birth. The mothers completed the questionnaires individually.

### The transition of Primiparas’ becoming mothers Scale

Mercer [28] suggested that the individual’s thought process regarding gaining the maternal role in becoming a mother should be changed. The maternal role involves coping with the child’s growth and changes in the environment as mothers constantly experience transitions throughout their lives. The scale is based on Mercer’s concept of the maternal role and is a tool developed to measure the transition of primiparous mothers to the maternal role. It consists of 30 items scored on a 5-point Likert-type scale (1 = “Not applicable” to 5 = “Applicable”). The total score varies between 30 and 150. There are reverse-scored items. The items are 1.2.3.4.5.6.7.8.9.10.26.29 on the scale. High scores from the scale indicate increased adaptation during the transition process of primiparas. The contents of the five dimensions are as follows: Factor I: Feeling of inadequacy in the maternal role, Factor II: What does child care mean to me?, Factor III: Feeling of mastery in fulfilling the role of mother, Factor IV: Relationship with one’s partner in child care, and Factor V: One’s own improving parenting perspective. The scale consists of 5 subdimensions, and the Cronbach’s α coefficient for each subdimension was 0.871 for Factor I, 0.870 for Factor II, 0.751 for Factor III, 0.767 for Factor IV, and 0.648 for Factor V [4].

### Translation of the transition of Primiparas becoming mothers Scale

The back-translation method was used to test the linguistic validity regarding the semantic equivalence of the scale’s items in the desired language [29]. The back-translation method is the most common method used to assess linguistic validity [30]. In this study, three translators translated the TPM-S from English to Turkish. An academic who speaks both languages ​​checked the translated version. The researchers revised the scale based on the academic feedback. Three fluent translators in both languages translated the Turkish version back into English. The researchers rechecked all translated versions, selected the items that best represented the dimensions in both languages and made some changes. Ten experts were consulted on TPM-S women’s health.

### Data analysis

The data obtained in the study were analyzed using SPSS (Statistical Package for Social Sciences) for Windows 26.0 and AMOS (Analysis of Moment Structures) 23.0. The sociodemographic characteristics of the mothers were analyzed using descriptive statistics. Percentages were used for categorical data on sociodemographic characteristics, and means and standard deviations were used for continuous data. The Davis technique was used to measure the level of expert agreement on the TPM-S items. The Kaiser‒Meyer‒Olkin (KMO) test was used to determine sample adequacy in the scale. Bartlett’s test of sphericity was used to determine whether the correlation was suitable for factor analysis. The root mean square error of approximation (RMSEA), comparative fit index (CFI), goodness-of-fit index (GFI), and Tucker‒Lewis index (TLI) were used for confirmatory factor analysis. Test-retest reliability was used to determine the reliability of the TPM-S, and Cronbach’s alpha reliability coefficient was used for internal consistency. The significance value was accepted as 0.05.

## Results

### Sample characteristics

Three hundred and five primiparous mothers were included in the study. The average age of the mothers participating in the study was 26.87 ± 4.62 years; 42.6% were high school graduates (*n* = 130), and 71.5% were not employed (*n* = 218). A total of 50.8% of the participants lived in the district (*n* = 155), 70.8% had a nuclear family (*n* = 216), and 52.8% were at the middle-income level (*n* = 161). A total of 25.6% of the mothers had a normal birth (*n* = 160) in the third postpartum month (*n* = 78) (Table 1).

**Table 1:** Participants’ demographic characteristics (*n* = 306)

Variables		n	%
Age (years old) ($\stackrel{-}{\varvec{X}}$±SS, 26.87 ± 4.62)	Under 27	148	48.5
	27 years and over	157	51.5
Educational background	Primary school	11	3.6
	Middle school	45	14.8
	High school	130	42.6
	University and above	119	39.0
Working status	I’m working	87	28.5
	I am not working	218	71.5
Place of residence	Village	47	15.4
	District	155	50.8
	Province	103	33.8
Family type	Nuclear family	216	70.8
	Extended family	89	29.2
Household income	Bad	74	24.3
	Medium	161	52.8
	High	70	23.0
Postnatal month	1	24	7.9
	2	35	11.5
	3	78	25.6
	4	63	20.7
	5	60	19.7
	6	45	14.8
Type of birth	Normal	160	52.5
	C-Section	145	47.5
Total		**305**	**100.0**

### Content validity

The Davis technique [31] was used to assess the scale’s content validity. To ensure the content validity and face validity of the scale, the opinions of academicians who are experts in the field of nursing/midwifery in Türkiye were consulted. The experts evaluated each item on the scale as follows:

- “Appropriate.”
- “The item should be slightly revised.“
- “The item should be seriously revised.”
- ” The item is not appropriate.”

The content validity index was calculated by dividing the number of experts who marked (a) and (b) each item by the total number of experts who expressed their opinions. The results showed that the experts had a high view of the interpretability/clarity and cultural appropriateness of the TPM-S items (CVI = 0.97). They agreed at the level of agreement and that there would be no change in any of the items. To ensure the clarity of the scale, 20 primiparous mothers were asked to complete the scale. None of the mothers reported any problems with the scale items. The 20 primiparous mothers included in the pilot study were not included in the main study.

### Construct validity

Before applying exploratory factor analysis, the Kaiser–Meyer–Olkin (KMO) test was used to test whether the sample size was suitable for factor analysis. The analysis revealed that the KMO value was 0.933. In line with this result, it was concluded that the sample adequacy was “sufficient” to conduct factor analysis. KMO values ​​between 0.5 and 1.0 are considered acceptable, while values ​​below 0.5 indicate that factor analysis is unsuitable for the dataset [32]. Additionally, when the Bartlett sphericity test results were examined, the chi-square value obtained was acceptable (χ2 (300) = 5286.273, *p* < 0.05; Table 2).

**Table 2:** Explanatory factor analysis results of the transition of primiparas becoming mothers scale

	Factors		Total Item Correlation
	F1	F2	
Item13	0.819		0.771
Item16	0.809		0.766
Item18	0.797		0.760
Item 23	0.791		0.759
Item 21	0.790		0.786
Item 28	0.773		0.742
Item 24	0.771		0.739
Item 30	0.751		0.744
Item 22	0.751		0.718
Item 19	0.737		0.724
Item 12	0.726		0.668
Item 27	0.723		0.708
Item 25	0.707		0.694
Item 15	0.703		0.674
Item 11	0.697		0.632
Item 20	0.697		0.670
Item 17	0.606		0.613
Item 3		0.823	0.764
Item 1		0.816	0.729
Item 4		0.809	0.766
Item 6		0.805	0.755
Item 5		0.780	0.720
Item 2		0.761	0.701
Item 7		0.757	0.687
Item 8		0.651	0.563
Reliability	0.952	0.911	0.935
Explained Variance (%)	38.338	20.938	59.276

KMO = 0.933; χ2(300) = 5286.273; Bartlett’s Test of Sphericity (p) = 0.000

### Exploratory factor analysis and factor naming

**Fig. 1:**
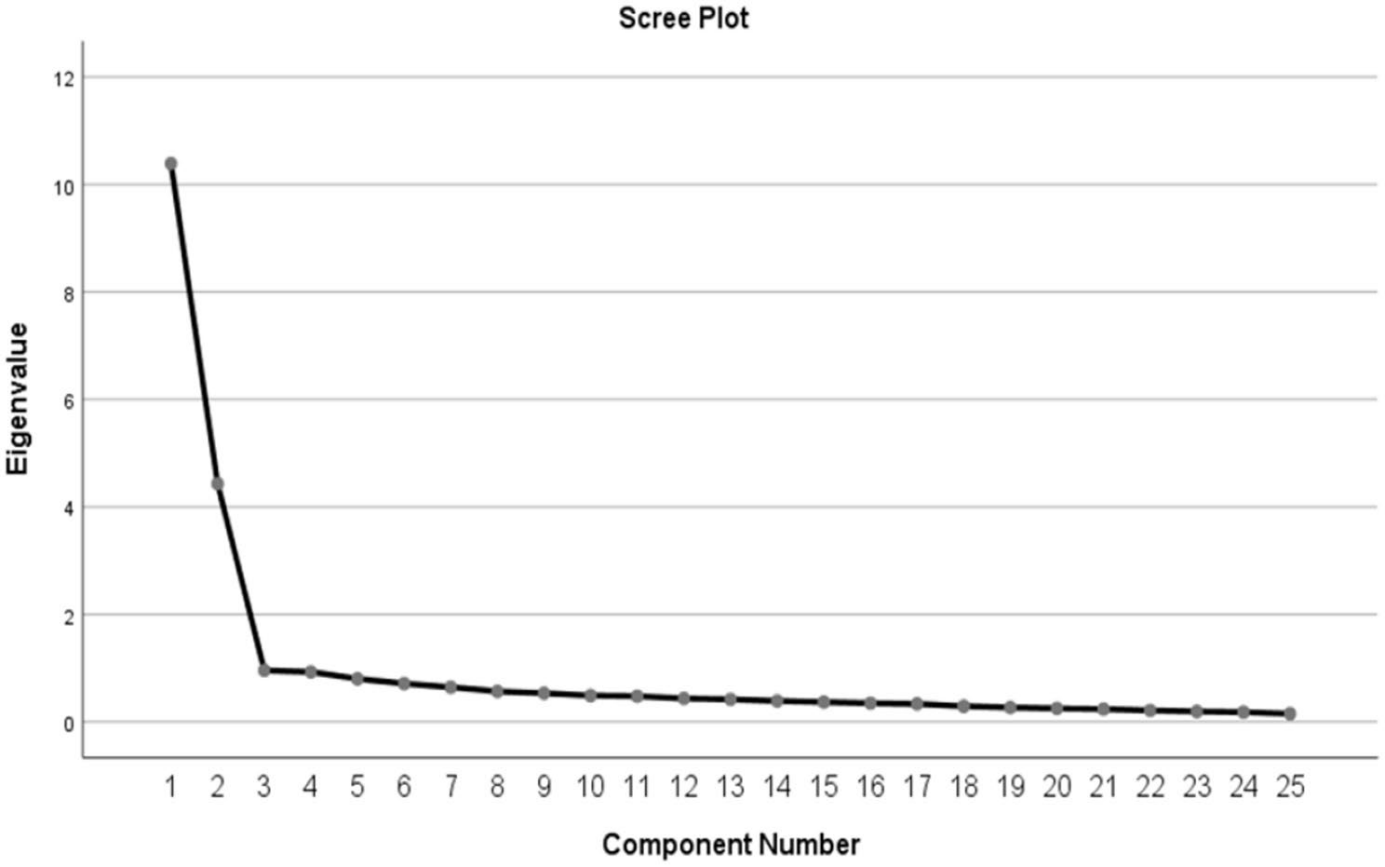
Scree plot for factor components of TPM-S

Factor loading describes the relationships between items and factors. There was no limitation on the number of dimensions when performing EFA. As a result of the analysis, the items with low factor loads, those that did not load on a theoretically significant dimension, those that loaded on more than one factor, and those with differences between factor loads of less than 0.01 were excluded from the analysis one by one, and the analyses were repeated. Thus, the final structure of the scale was revealed (Table 2). The factor loadings of the TPM-S items ranged from 0.58 to 0.81, and some items were removed from the scale because they were below 0.40. The criterion for the items to remain on the scale was that their factor loadings were more than 0.40 [33]. Başol [34] stated that the discrimination power of an item, expressed as the coefficient of determination or validity of the measured feature, should have values ​​of 0.40 and above. In the original scale, 30 items were grouped into five subscales. In the explanatory factor analysis conducted to reveal the factor pattern of the scale and based on the scree plot, five items with low factor loads were removed from the scale (item 9, item 10, item 14, item 26, item 29). The remaining 25 items were collected in two subdimensions (item 9, item 10, item 14, item 26, item 29; Fig. 1). Factor I was named ‘sense of mastery in fulfilling the role of mother’ and consisted of items related to the awareness of understanding the demands of the child and starting to act like a mother. Factor II was defined as a ‘feeling of inadequacy in the maternal role’ and consisted of items related to the inadequacy and lack of self-confidence in one’s parenting and the inability to fulfill the maternal role fully. These factors explained 59.276% of the total variance (Table 2). In multifactor designs, it is considered sufficient if the explained variance is greater than 50% [35]. When the correlations between variables are examined, the factor loadings of the items are above 0.40, and all correlation relationships are significant (Table 2). The factors were rotated with the Varimax rotation process [36].

### Confirmatory factor analysis

The standardized values ​​(factor loadings), model goodness of fit, validity, and composite reliability values ​​of the models tested with first-level CFA are given in Table 3. The CR and AVE values ​​of the factors are presented in the table. The table shows that the CR values ​​are above 0.70. This CR indicates that the scales are reliable. All the AVE values ​​of the factors are above 0.50. This AVE shows that the convergent validity of the scales was achieved.

**Table 3:** First level multi-factor confirmatory factor analysis results of the scale and item analysis results

Factors	Items	Factor Load	Standart Deviation	t	p	AVE	CR	t (%27 Over-Under)	p (%27 Over-Under)
F1	Item13	0.777	-	-	-	0.54	0.95	-20.147	0.000***
	Item16	0.787	0.066	15.004	***			-20.252	0.000***
	Item18	0.786	0.066	14.962	***			-21.214	0.000***
	Item 23	0.765	0.050	18.614	***			-19.108	0.000***
	Item 21	0.805	0.069	15.434	***			-23.334	0.000***
	Item 28	0.753	0.068	14.191	***			-19.101	0.000***
	Item 24	0.768	0.066	14.549	***			-20.435	0.000***
	Item 30	0.755	0.067	14.226	***			-27.047	0.000***
	Item 22	0.743	0.066	13.966	***			-18.956	0.000***
	Item 19	0.745	0.070	14.019	***			-22.268	0.000***
	Item 12	0.684	0.068	12.647	***			-18.814	0.000***
	Item 27	0.712	0.072	13.245	***			-17.732	0.000***
	Item 25	0.698	0.073	12.951	***			-20.272	0.000***
	Item 15	0.692	0.073	12.825	***			-19.462	0.000***
	Item 11	0.647	0.070	11.852	***			-18.346	0.000***
	Item 20	0.693	0.065	12.838	***			-18.288	0.000***
	Item 17	0.636	0.074	11.608	***			-17.848	0.000***
F2	Item 3 R	0.810	-	-	-	0.57	0.91	-21.388	0.000***
	Item 1 R	0.772	0.064	14.965	***			31.791	0.000***
	Item 4 R	0.811	0.060	16.001	***			-22.144	0.000***
	Item 6 R	0.795	0.064	15.565	***			-23.672	0.000***
	Item 5 R	0.760	0.064	14.663	***			-19.901	0.000***
	Item 2 R	0.743	0.061	14.228	***			-19.805	0.000***
	Item 7 R	0.713	0.062	13.492	***			-23.952	0.000***
	Item 8 R	0.588	0.067	10.655	***			-14.801	0.000***

***p < 0.05 R: Reverse Item

**Fig. 2:**
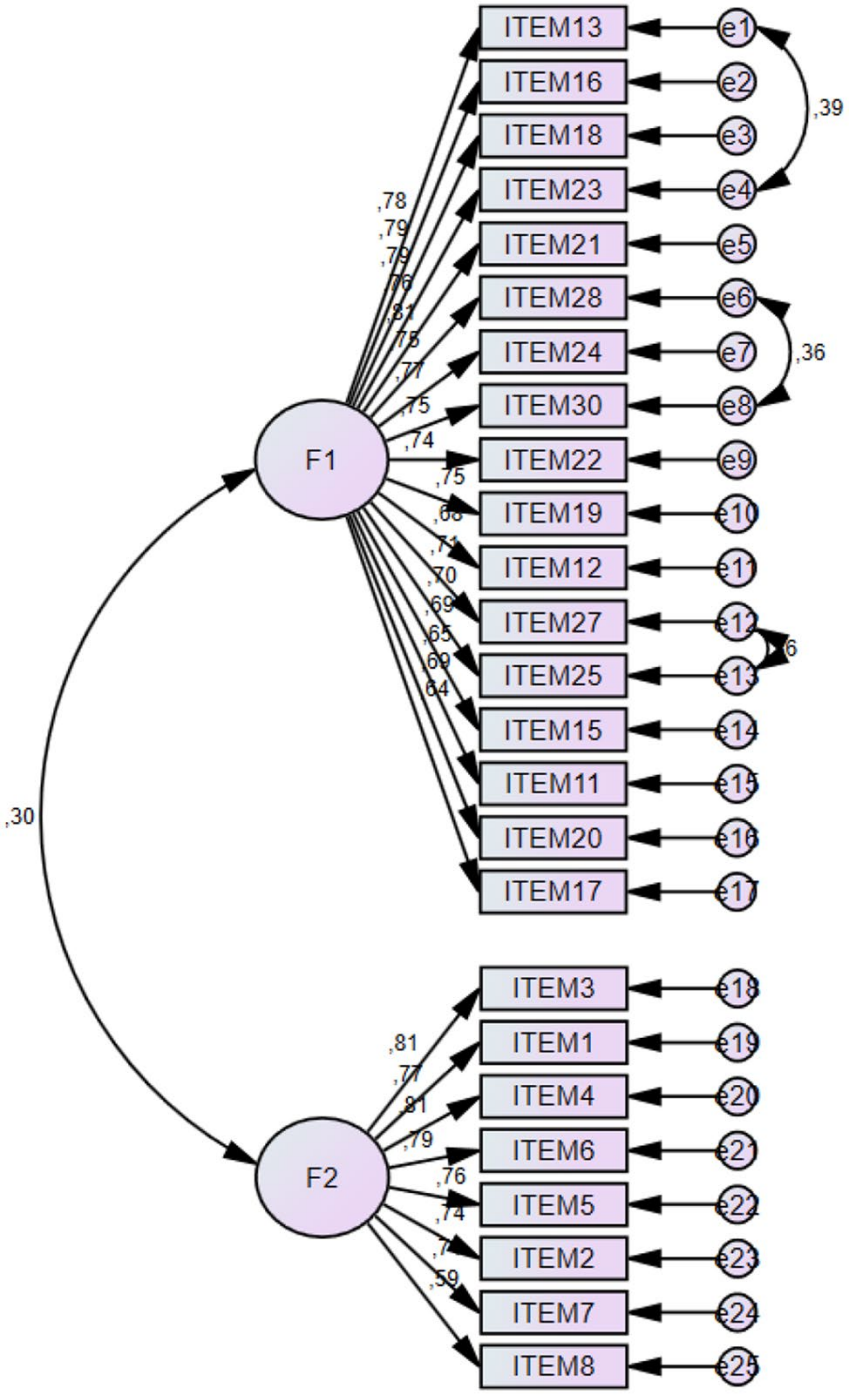
Confirmatory factor analysis

According to the confirmatory factor analysis, it was determined that the 25 items that make up the scale were related to the 2-dimensional scale structure (Table 4). Improvements are being made to the model. While improving, variables that reduce fit were identified, and a new covariance was created for those with high covariance between residual values. Afterwards, in the renewed fit index calculations, the accepted values ​​of the appropriate indices are shown in Table 4. We found that CMIN/DF = 3.022. The RMSEA is an index that evaluates fit as a function of degrees of freedom; higher values ​​indicate poorer fit, and a value below 0.08 indicates an acceptable fit [37]. We found that the RMSEA was 0.079 (Table 4). In this study, the CFI was 0.894, the GFI was 0.817, the NFI was 0.850, and the TLI was 0.882 (Fig. 2).

**Table 4:** Fit indices and acceptable values ​​in confirmatory factor analysis and pearson product-moment correlation (*n* = 40)

	Values Found	Acceptable Values
CMIN/DF	3.022	≤ 5
RMSEA	0.079	≤ 0.80
GFI	0.817	≥ 0.80
CFI	0.894	≥ 0.80
TLI	0.882	≥ 0.80
IFI	0.894	≥ 0.80
RFI	0.834	≥ 0.80
NFI	0.850	≥ 0.80
SRMR	0.067	≤ 0.10
Total Scores	**R**	p
Scale Test	1	0.000^**^
Scale Retest	0.961	0.000^**^

** Correlation is significant at the 0.01 level (2-tailed)

### Internal consistency reliability

Test-retest reliability was used to determine the internal consistency of the TPM-S. The scale was applied to 40 primiparous mothers who returned for routine postpartum outpatient clinic control (first measurement). The retest was applied to the same 40 primiparous mothers who came to FHCs to vaccinate their babies on the 30th day (second measurement). The Pearson product-moment correlation coefficient was used to determine the correlation between the test and retest scores used to measure the internal consistency of the scale, and there was a significant correlation between the scores ([27, 38], *p* < 0.01, Table 4). In addition, item analysis based on subsupergroups was conducted to test the internal consistency reliability. The findings are summarized in Table 3. As a result of the comparison, there is a significant difference between the averages of the lower and upper group item scores at the *p* < 0.05 level for all items for each sub dimension. Based on this, the scale’s subdimensions are distinctive in measuring the desired quality.

### Reliability results

The Cronbach’s alpha value of the scale is 0.93, which shows that the scale is quite reliable. Among the subscales of the scale, the Cronbach’s alpha of Factor I was 0.95, and the Cronbach’s alpha of Factor II was 0.91 (Table 2). This shows that the subdimensions of the scale are also quite reliable. There was a significant correlation between the total scale and item scores of the scale (*p* < 0.001).

## Discussion

We examined the psychometric properties of the version of the TPM-S adapted to Turkish culture. In Türkiye, measurement tools that determine the maternal role of primiparous mothers and their transition to motherhood are inadequate, and no measurement tool measures the transition to motherhood. This study is the first to psychometrically test the TPM-S among primiparous mothers in Türkiye. The psychometric results of the adapted scale were consistent with the results of the original scale [4]. Although the study was close to the original scale despite the lack of subscales and items, it could not be compared or discussed with other studies because the scale was not adapted to different cultures.

Research results show that the TPM-S is a valid and reliable measurement tool for primiparous mothers in Turkey. The TPM-S is a measurement tool that can be applied internationally and translated into other languages. The Cronbach’s alpha value of the TPM-S was 0.93, exceeding the recommended value [39, 40]. The Cronbach’s alpha of Factor I was 0.95, the Cronbach’s alpha of Factor II was 0.91, and our results were similar to those of the original TPM-S. The Cronbach’s alpha values ​​corresponding to the factors in the original scale were 0.75 and 0.87 [4]. Since this is the first study to determine the validity and reliability of the TPM-S on primiparous mothers from different cultures, further multicenter validation studies should be conducted with larger samples from various cultures to confirm our results.

Test-retest tests were used to assess the reliability and internal consistency of the TPM-S. There was a significant correlation between the two tests performed within a specific period (*r* = 0.96, *p* < 0.001). A parallel form was used instead of a test-retest to check the reliability of the original scale. Since there is no other research on the TPM-S for primiparous mothers, further validity and reliability studies are needed to test our results. In the item analysis based on lower and upper groups to test internal consistency, the adapted scale is distinctive in measuring the desired quality, as there was a difference in the significance level between the lower and upper group item scores for each subdimension.

Exploratory and confirmatory factor analyses were conducted to determine the scale’s construct validity. KMO was 0.93, and the Bartlett test of sphericity was significant (*p* < 0.001), indicating that the minimum number of participants recommended for each test was provided for the sample to be considered sufficient [41, 42] and that there was an adequate correlation between the variables for factor analysis. Exploratory factor analysis (EFA) was used to examine the factor loadings of the TPM-S items. Factor loading describes the relationships between items and factors. Başol [34] stated that the discrimination power of an item, expressed as the coefficient of determination or validity of the measured feature, should have values ​​of 0.40 and above. Five items with factor loadings below 0.40 were removed from the scale. Five subdimensions in the original scale were reduced to two subdimensions, and 30 items were reduced to 25 items [4]. Items with factor loads of 0.32–0.44 indicate poor, items with 0.45–0.49 indicate fair, items with 0.50–0.62 indicate good, items with 0.63–0.70 indicate very good, and items with ≥ 0.71 indicate excellent [43, 44]. The factor loadings of the remaining items of the TPM-S were between 0.58 and 0.81. In the original scale, the common factor loadings of each item were > 0.40, suggesting that each item was highly related to the identified factor [4].

The CR value of the model tested with first-level CFA showed that the scale was at a sufficient level of reliability, and the AVE value indicated that the scales achieved convergent validity. CR values ​​must be greater than 0.70 [45], and AVE values ​​must be 0.50 or greater [46]. The AVE and CR values ​​were acceptable.

CFA was performed to assess the construct validity of the original scale when it was adapted to another culture. The aim is to determine the similarities and differences between the adjusted and original scales. Degrees of freedom are an essential criterion for the chi-square test. The chi-square value is the most basic measurement used to test the general suitability of a model. When determining the level of fit of the model with the data, multiple model fit indices should be examined. This model is constructed with degrees of freedom and a chi-square test. The chi-square/degrees of freedom ratio is used as the appropriateness criterion, and a ratio less than 5 indicates an acceptable value for the scale [47].

For the RMSEA, a measure of the discrepancy between the population and the observed covariance per degree of freedom, a value below 0.08 indicates an acceptable fit [37, 48]. The study found the RMSEA to be at an acceptable level.

CFI obtains results by comparing the null model with the sample covariance matrix, taking values ​​between 0 and 1. The model’s fit increases as it approaches one and is a measure of model fit relative to the fit of an independent model that is assumed to provide a poor fit to the data [49]. In this study, the CFI value indicates the suitability of the model. The GFI is basically the result of the ratio of model covariances and variances to the measured variances and covariances. In short, it is the proportional comparison of the real and the modeled data [50]. The GFI statistic takes values ​​between 0 and 1 and moves inversely proportional to the degrees of freedom. Therefore, the ratio of sample size to degrees of freedom tends to increase as the sample size increases [51]. Traditionally, a threshold value of 0.90 is recommended, but when small sample sizes and factor loadings are found to be low, an evaluation up to a threshold value of 0.95 can be made [51].

The GFI in the study was 0.817. A GFI value < 0.90 indicates a relatively more minor observable variable and a smaller GFI [52]. Therefore, the number of items may have affected the results of this study. However, increasing the number of items will cause the response rate to decrease and the number of people leaving the survey unfinished and inappropriate responses to increase. Finally, the normalized fit index (NFI) ranges from 0 to 1, with an NFI greater than or equal to 0.90, indicating an acceptable fit [53]. When necessary, as the number of items and the sample increase, the acceptable value may be > 0.80 [54], and the NFI value in this study was at an acceptable level. Studies with larger samples are needed to better verify and investigate the model fit of the scale. However, NFI may provide less fit than that found in models studied with (small) samples below 200 [54].

To eliminate this problem, the TLI is used as an alternative to the NFI. There are many different opinions in the literature regarding the TLI threshold value. A threshold value of TLI > 0.80 is acceptable [49]. The TLI value in the study was 0.88, and the model fit well in this study, which was conducted with 305 samples.

### Scale practicality

The current study adapted the original scale to Turkish society based on interviews with Turkish primiparas up to 6 months postpartum while adjusting to life as a mother. Unlike the original scale, the adapted scale was introduced to Turkish society and the literature with 25 items and two subdimensions. While scoring according to the overall scale and its subdimensions, it can be used to reveal which part of life as a mother the participants included in the study were accustomed to and to what extent they became accustomed to it.

In addition to giving high or low scores to specific parameters, the scale is essential for awareness of the individual’s place in the motherhood role. With this scale, mothers will be able to determine at what stage they are in parenthood. In line with the original scale’s recommendations, the scores obtained from the scale and its subdimensions were evaluated in an adaptation study made for Turkish society. It was found that as the total score of the subdimensions and the total score of the overall scale increased, the adaptation of primiparous mothers in the transition to motherhood increased.

### Strengths and limitations

The first of the most vital aspects of this study is that a valid and reliable measurement tool that researchers and health professionals in Türkiye use for primiparous mothers has been adapted to Turkish society and culture. Second, the scale we adapted is the first of its kind since there is no measurement tool to determine the transition process of primiparous mothers to motherhood in Türkiye. The most important limitation was that since no equivalent scale was developed for primiparous mothers, reliability analysis could not be performed, and the test-retest method was used instead.

## Conclusion

The study’s original English version of the TPM-S was translated into Turkish and administered to 305 primiparous mothers. The translated version had acceptable goodness-of-fit values ​​and a high reliability coefficient. The TPM-S is a valid and reliable measurement tool that can be used to determine the transition experiences of primiparous mothers to motherhood. Health professionals and researchers can use this scale to identify primiparous mothers who need support in parenting and thus effectively support primiparous mothers who perceive themselves as inadequate in childcare and the maternal role. Health professionals can routinely apply the form to primiparous mothers in the first six months after birth to determine their transition to motherhood. They can also consider education and support initiatives in the care practices they plan to plan for first-time mothers.

## Data Availability

The corresponding author, upon reasonable request, will provide data supporting the findings of this study.
